# Molecular and Epidemiological Analyses of Sheeppox Outbreaks in Russia from 2013 to 2021

**DOI:** 10.1155/2023/8934280

**Published:** 2023-10-10

**Authors:** Alexander Sprygin, Kseniya Shalina, Antoinette van Schalkwyk, Ali Mazloum, Sergey Shcherbinin, Alena Krotova, Olga Byadovskaya, Larisa Prokhvatilova, Ilya Chvala

**Affiliations:** ^1^Federal Center for Animal Health, Vladimir, Russia; ^2^Agricultural Research Council-Onderstepoort Veterinary Institute, 100 Old Soutpan Road, Onderstepoort 0110, South Africa; ^3^Department of Biotechnology, University of the Western Cape, Robert Sobukwe Road, Bellville 7535, South Africa

## Abstract

Sheeppox (SPP) is a transboundary infectious disease that affects sheep and goats, leading to significant losses in countries with extensive small ruminant breeding programs. While sporadic SPP outbreaks have been observed in the Russian Northern Caucasus and the Far East, the number of SPP outbreaks in the Russian Central and Far East regions has increased recently. However, there is a lack of epidemiological data on SPP virus (SPPV) in the Northern latitudes. In this study, we conducted epidemiological and genetic analyses of SPP outbreaks in Russia from 2013 to 2021 using whole-genome sequences of five selected isolates. The epidemiological data revealed an elevated risk of SPP outbreaks during the summer months, although outbreaks were not limited to this period. A phylogenetic analysis of the whole-genome sequences of selected SPPVs obtained directly from clinical samples revealed a unique cluster of SPPV isolates circulating in Central Russia, which was related to previous isolates from the European part of Russia. In contrast, SPPVs obtained from the Russian Far East showed genetic similarity to isolates detected in Southeast Asia. The whole-genome sequences demonstrated that the reported outbreaks were not related to the NISKHI vaccine strain, which was used to combat lumpy skin disease and SPP in Russia. Based on the study findings and GenBank data, we propose a novel cluster designation system for SPPV genomic sequences.

## 1. Introduction

Sheeppox (SPP) is a transboundary infectious disease of sheep and goats and is listed as a reportable disease by the World Organization for Animal Health (WOAH). Its etiological agent, SPP virus (SPPV), belongs to the genus *Capripoxvirus* and the family *Poxviridae* [1, 2]. SPP outbreaks cause considerable economic damage in affected countries and impact global food security [3, 4]. SPP was previously thought to affect only sheep, but it has now been shown to infect goats as well [5]. This disease severely limits animal productivity, posing a threat to poor countries where sheep rearing is culturally significant [6].

During host infection, SPP manifests as nasal discharge, pyrexia, rhinitis, conjunctivitis, and classical pock lesions on the skin and mouth with necrosis. Pock lesions cause irreversible skin damage, resulting in scarring and devaluing the hide and wool quality [7]. In contrast to lumpy skin disease virus (LSDV) in cattle, which has high morbidity and low mortality rates, SPP is associated with high morbidity and mortality rates [8].

Poxviruses exhibit distinct host-cell specificity that may differ from their *in vivo* host range [9]. *Capripoxviruses*, which were previously thought to be restricted to a single host species, with names corresponding to the hosts from which they were obtained (such as LSDV in cattle, SPPV in sheep, and goatpox virus (GTPV) in goats), are not limited to a single host, as SPPV and GTPV infections have been reported in sheep and goats and LSDV in antelopes and giraffes [8, 10–13]. SPPV causes mild clinical symptoms in goats and severe clinical disease in sheep, whereas GTPV causes severe clinical disease in goats and mild clinical symptoms in sheep [14, 15]. Virus culture on different cell lines indicated that SPPV causes cytopathic effects similar to other *Capripoxvirus* strains, such as cell rounding, aggregation, and detachment from the surface [16–19].

SPPV is endemic to Northern and Central Africa, the Middle East, Europe, and Asia, where sheep play a crucial role in large commercial, small-scale, and subsistence farming practices [20–23]. In the Russian Federation (RF), SPP outbreaks have occurred sporadically in the Far East and Northern Caucasus, where sheep raising holds significant cultural importance [24]. From 2003 to 2012, 13 SPP outbreaks were reported in the RF, which increased to 72 in the last 10 years (https://web.oie.int/hs2/zi_pays.asp?c_pays=162&annee=2003, https://wahis.woah.org/#/event-management). Understanding the molecular evolution of SPPVs and the factors driving these outbreaks can help control and mitigate the spread of the disease in specific regions. Since epidemiological data on SPPV in the Northern latitudes are lacking, we analyzed SPPV outbreaks based on WOAH reports and performed whole-genome sequencing of selected SPPVs. The genetic relationship among strains obtained from outbreaks in various regions of the RF between 2013 and 2021 was determined.

## 2. Materials and Methods

### 2.1. Data Collection

Data on the epidemiological situation of SPP in the RF were retrieved from annual reports submitted to the WOAH (https://wahis.woah.org/#/event-management). The data included information on the coordinates of SPP outbreaks, the number of outbreaks during the study period, and the average distance between outbreaks. These locations were primarily in the Central Federal District, a region with a high density of outbreaks. The spread of SPPV in the RF from 2013 to 2021 was analyzed by mapping the number, location, and average distance between outbreaks using the ArcGIS program (Esri, USA).

### 2.2. Samples

**Figure 1:**
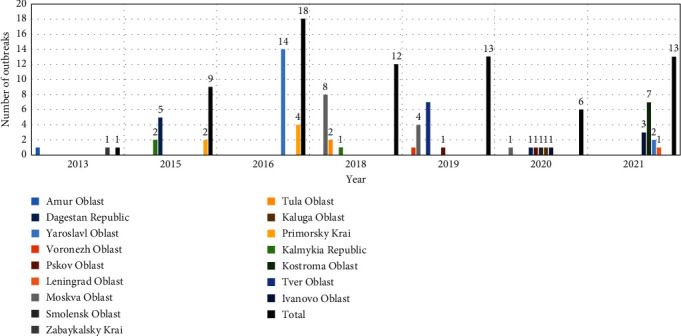
Diagram showing the total number of SPP outbreaks reported annually in different regions of the RF from 2013 to 2021.

**Figure 2:**
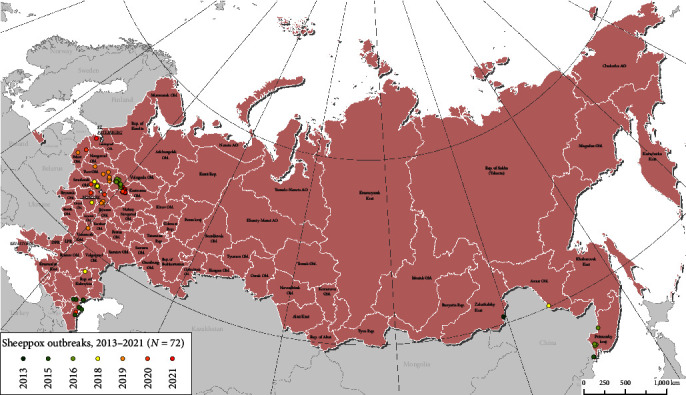
SPP-affected regions in the RF from 2013 to 2021.

Five virulent SPPV samples, representing a wide geographic distribution, were obtained from active outbreaks between 2018 and 2020 for complete genome sequencing. The remaining samples discussed in Figures 1 and 2 were not available for full genome sequencing. Scabs were aseptically collected from dead animals with clinical symptoms of SPP. Sequencing was performed with DNA extracted directly from clinical samples without virus isolation or propagation on cell culture. The metadata for each sample are summarized in Table 1.

### 2.3. Sequencing

Total DNA was extracted using Trizol (Invitrogen) following the manufacturer's instructions. Prior to DNA extraction, tissue samples were crushed with a sterile grinder, and a 10% (v/w) suspension with phosphate-buffered saline (Invitrogen, USA) was prepared.

Then, 200 ng of purified DNA was fragmented into 100–700 bp fragments, with a peak distribution between 250 and 300 bp, using a Covaris ME220 focused-ultrasonicator (Covaris, USA) following the manufacturer's instructions. About 300 bp fragments were purified using magnetic beads (SPRI), and 25 ng of DNA was processed according to the MGIEasy Universal DNA Library Prep Set (MGI Tech, China) protocol. The latter entails blunt-end polishing of DNA fragments and ligation to 10-bp single-end index adapters (MGI Tech, China).

The DNBSEQ-G400 platform (MGI Tech) and pair-end 150 sequencing protocol were used to generate datasets. Each sample yielded datasets with 10–100 million paired reads.

### 2.4. Bioinformatics

All reads obtained were mapped against the NISKHI SPPV sequence (GenBank accession number: AY077834) using CLC Genomics v9 Workbench (Qiagen, Germany). The average coverage of the mapped reads ranged between 16 and 497 across the samples, resulting in the generation of a single consensus sequence per sample. These consensus sequences were deposited in GenBank under the accession numbers OQ434235–OQ434239.

To investigate the phylogenetic relatedness, a total of 16 whole-genome sequences representing various vaccine strains and field isolates were aligned with the five newly generated consensus sequences. This alignment was performed using CLC Genomics Workbench v9. The aligned sequences were then used to construct a phylogenetic tree using the maximum likelihood model with General Time Reversal + 4 and 100 bootstrap iterations in Mega X [25]. The alignment was also used to identify individual single nucleotide polymorphisms (SNPs) between the SPPV sequences.

## 3. Results

### 3.1. Epidemiological Situation Analysis

Between 2013 and 2021, a total of 72 SPP outbreaks were reported in 16 regions of the RF. However, no outbreaks were reported in 2014 and 2017, while a single outbreak was reported in 2013. The majority of outbreaks occurred in 2016, with a total of 18 outbreaks. In that year, 14 (78%) of the outbreaks were reported in the Yaroslavl Oblast (Figure 1). In contrast, the outbreaks in 2020 were reported from six different geographical regions, including Moskva, Dagestan, Pskov, Smolensk, Kaluga, and Ivanovo Oblasts (Figure 1).

Before 2015, SPP outbreaks were mainly limited to the Eastern and Southern regions of the RF. However, since 2016, the majority of outbreaks have been reported in the Central Federal District (Figure 2).

**Figure 3:**
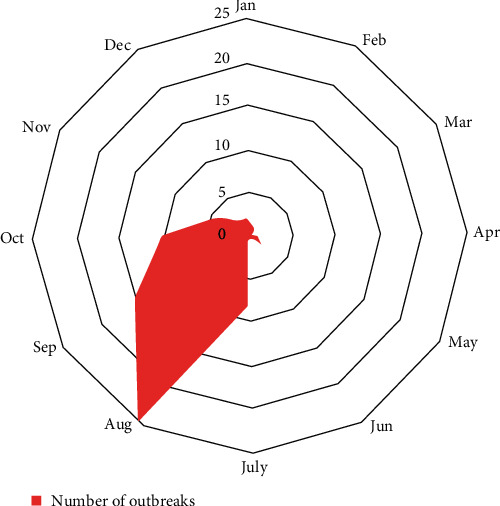
Number of SPP outbreaks per month in the last 10 years.

During 2013–2021, the majority of SPP outbreaks (58) occurred between July and October, with the highest number of outbreaks in August and September (*n* = 35 or 60%) (Figure 3). This indicates that the warmer months have a greater impact on the transmission of SPP than the colder months. The average distance between outbreaks was calculated to be 27 km using the mean nearest neighborhood method in the ArcGIS program for the European part of the RF.

### 3.2. Phylogenetic Analysis of Full Genomes

**Figure 4:**
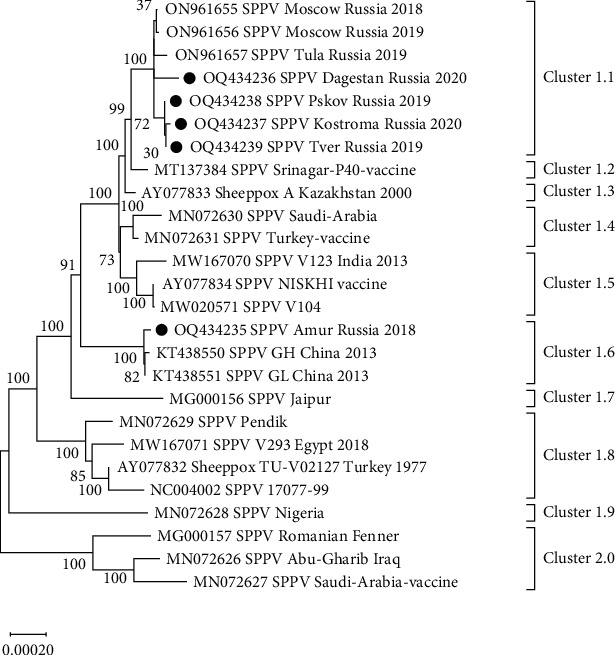
Maximum likelihood phylogenetic tree illustrates the relationship among all available SPPV sequences from GenBank and those generated in this study. The latter are designated with black dots.

In addition to the five whole-genome sequences generated in this study, 21 additional sequences were obtained from GenBank, including three SPPVs from the RF in 2018 and 2019 [26]. Based on phylogenetic comparisons of 28 SPPV whole-genome sequences, 10 phylogenetic distinct clusters were proposed (Figure 4), with sequences divided into two major clusters. Cluster 1 was divided into nine subclusters (1.1–1.9) and represented all field isolates, except for subclusters 1.2, 1.4, and 1.5 that represented Sprinagar-P40 vaccine, Turkey vaccine, and NISKHI vaccine, respectively. Cluster 2.0 had only one subcluster and included Abu Gharib Iraq isolate and Saudi Arabia vaccine (Figure 4).

Cluster 1.9 was monophyletic, with only one SPPV from Nigeria (MN072628), whereas Cluster 1.8 included four isolates from Turkey: 10-700-99 (NC004002), TU V0-2127 (AY077832), V293 (MW167071), and Pendik (MN072629). Cluster 1.6 comprised two isolates from China in 2013 (KT438551 and KT438550) and one isolate (Amur/2018) from Russia. Clusters 1.3 and 1.2 were monophyletic, representing strains A from Kazakhstan (AY077833) and Srinagar P40 from India (MT137384), respectively (Figure 4). Cluster 1.1 included samples collected from Central Russia that were either generated previously (ON961655–ON961657) or during this study (Figure 4).

Cluster 2.0 included vaccine strains from Abu Gharib and Saudi Arabia (MN072626 and MN072627), whereas Cluster 1.5 included the vaccine strain NISKHI (AY077834) as well as V123 and V104 (MW167070 and MW167071) of unknown origin (Figure 4). Cluster 1.4 included vaccine strains from Saudi Arabia and Turkey (MN072630 and MN072631) (Figure 4).

Table S1 presents the pairwise comparison of nucleotide differences and percentage sequence similarity between the sequences. The new sequence, Amur/2018, differed by 13–17 bp from the two isolates from China in 2013, with 99.99% sequence identity (Table 2). In contrast, the four newly generated sequences in Cluster 1.1 shared 99.93%–100% sequence identity. Among the eight SPPV sequences from Russia, the NISKHI vaccine strain, and the two 2013 isolates from China, a total of 41 nonsynonymous, 69 synonymous, and 52 SNPs were detected within the intragenic regions (Table 2). In addition, compared with the seven other sequences from Russia, Amur/2018, the NISKHI vaccine, and the two isolates from China shared eight nonsynonymous SNPs (Table 2). Moreover, compared with the NISKHI vaccine and samples from Russia, Amur/2018 and the isolates from China had 22 unique nonsynonymous SNPs (Table 2). Furthermore, Dagestan/2020 had 11 unique nonsynonymous SNPs compared with all available SPPV sequences (Table 2).

## 4. Discussion

This is the first study to investigate the molecular epidemiology of SPP in the Northern latitudes using both epidemiological and genomic data [27]. Previous studies primarily focused on occasional reports of whole-genome sequencing of individual isolates, limiting our understanding of global SPP distribution and evolution [25, 26, 28].

In the RF, SPP cases were previously limited to sporadic outbreaks in the Northern Caucasus and regions near China and Mongolia in the Far East [24]. SPP is considered endemic in China and Mongolia due to their traditional sheep-raising practices, implying that SPP outbreaks were historically limited to Russian border regions [29]. Due to the limited number of outbreaks in the RF, the only available SPPV genetic data in the RF belonged to strains obtained from outbreaks in the country's border regions and analyzed by sequencing the *P32* locus, the GPCR, and PRO30 gene regions. However, a recent study performed the first whole-genome sequencing of SPPV from the Central region of the RF, revealing a novel phylogenetic cluster [24, 26].

This study analyzed 72 SPP outbreaks in the RF over a 9-year period. Of these, 16 outbreaks occurred in the Russian Central region, which is far from any border regions that are normally at risk of transboundary outbreaks. Outbreaks in the Russian Central region have not been reported before [26]. Due to the significant implications of these new outbreaks, additional investigations into the reasons for northward incursions into the central regions are required. SPP outbreaks were more prevalent during summer months than during winter months, possibly due to the contagious nature of SPPV and the mode of virus transmission [30, 31]. When sheep are confined to stall keeping during the winter in Russian Central and Northern regions, important mechanisms of SPPV infection transmission are mitigated by subsequent isolation and movement control. These include joint grazing with infected animals; infection via a common watering hole; virus introduction via transport of infected animals, feed, or bedding; and mechanical insect vectors, although the latter has been shown to play only a minor role [32]. All Capripoxviruses, including the novel recombinant LSDVs, have been shown to transmit via direct and/or indirect contact in either warm or cold conditions [33–35].

In countries with tropical and subtropical climates, such as Ethiopia, SPP outbreaks typically occur during wet months from the end of summer (August) to the dry, cold spring season, with a peak in November [36]. Reports from countries with warmer climates without snow, such as Israel and India, demonstrated that SPP outbreaks occurred in the winter and spring months [37–39]. In the Mediterranean, Greece reported an increase in SPP outbreaks from summer to January [40], whereas Tunisia has outbreaks throughout the year, with major peaks in autumn and winter [41]. In contrast, Mongolia has reported SPP outbreaks from September to November [42], which is consistent with the Amur/2018 strain detected during the freezing conditions of December in Russia (Figure 1).

This study is the first to investigate the genetic diversity of SPPVs from Northern latitudes using whole-genome sequencing. Currently, GenBank contains 21 SPPV whole-genome sequences from India, China, Nigeria, Turkey, Saudi Arabia, and Kazakhstan. However, more genomic data analysis is needed to understand the SPP epidemiological situation. In this study, SPPVs from outbreaks over 4 years (2018–2022) and a distance of 2,144 km from Dagestan to Pskov demonstrated genetic variability, forming a unique phylogenetic cluster (1.1) consisting only of samples from Russia (Figure 2). These Russian isolates suggest the introduction of a common ancestor with strains from India and Kazakhstan, which has evolved and is circulating in the Russian Central region. These SPPVs may have been circulating in the region for decades in sheep or other hosts. *Capripoxvirus* genomes have low mutation rates but are susceptible to accelerated genetic shifts due to recombination [26, 35, 43]. In contrast, previous research on single locus (*P32*) typing found no variations among the isolates from India or GTPVs over a 70-year period [44]. Since Roy et al. [44] focused on a conservative gene target, significant variation is not expected, and studies involving a single gene region provide limited information.

In the present study, the genomes showed nonsynonymous mutations compared with the attenuated NISKHI strain (Table 2). Each new sequence had unique SNPs that resulted in amino acid changes throughout the genome. However, we were unable to determine the specific effects of these mutations in this study. It is crucial to monitor these mutations for molecular epidemiological investigations of SPPV to identify possible patterns of purifying selection. In future studies, analyzing growth curves of virulent isolates would be the initial step.

Interestingly, apart from the three previously published sequences from Central Russia, no additional publications on the whole-genome analysis of SPPV in Russia, Central Asia, or Northern latitudes were found [26]. Only one study was conducted, which sequenced an isolate from Zabajkalskij Kray at *P32* to compare its similarity to isolates from China [24]. Our study expanded on the work of Krotova et al. [26] by providing a broader sequence context, revealing the presence of two SPPV lineages, 1.6 and 1.1, in the Far East and European parts of Russia, respectively. Some variation was observed in Cluster 1.1 isolates, with the Dagestan/2020 isolate from the North Caucasus showing nonsynonymous SNPs that differed from the rest (Table 2).

## 5. Conclusions

In conclusion, the SPPV epidemiology was investigated for the first time using outbreak metadata from WOAH and whole-genome sequencing. Although outbreaks continue to occur in frosty winters, the warm season is important for the transmission of SPPV in Northern latitudes. In addition, two genetic lineages were revealed: the dominant 1.1 and 1.6. These findings provide a basis for understanding SPP epidemiology in Eurasia and serve as a starting point for tracking the molecular evolution of SPPV within the proposed cluster designation.

## Figures and Tables

**Table 1:** Summary of metadata pertaining to the sequenced SPPV samples.

Sample name	Region in the RF	Submission date	Sample type	GenBank accession number
Amur	Amur Oblast	2018	Scabs	OQ434235
Pskov	Pskov Oblast	2019	Scabs	OQ434238
Tver	Tver Oblast	2019	Scabs	OQ434239
Kostroma	Kostroma Oblast	2020	Scabs	OQ434237
Dagestan	Republic of Dagestan	2020	Scabs	OQ434236

**Table 2:** Nonsynonymous single nucleotide polymorphisms were detected among eight sequences from Russia, China, and the NISKHI vaccine.

Open reading frame (position of predicted amino acid in translated protein)	Amur/2018	Dagestan/2021	Pskov/2019	Twer/2019	Kostroma/2020	ON961655/Moscow/2018	ON961657/Tula/2019	ON961656/Moscow/2019	AY077834/NISKHI- vaccine	KT438551/GL/China/2013
SP008 (274)	L	F	F	F	F	F	F	F	L	L
SP008 (233)	T	S	S	S	S	S	S	S	T	T
SP009 (22)	N	I	I	I	I	I	I	I	I	N
SP009 (9)	I	L	L	L	L	L	L	L	L	I
SP010 (155)	T	P	T	T	T	T	T	T	T	T
SP012 (177)	D	E	D	D	D	D	D	D	D	D
SP012 (165)	S	C	S	S	S	S	S	S	S	S
SP016 (40)	N	T	N	N	N	N	N	N	N	N
SP017 (166)	F	L	L	L	L	L	L	L	L	F
SP020 (6)	S	A	A	A	A	A	A	A	S	S
SP028 (265)	T	A	A	A	A	A	A	A	A	T
SP033 (144)	S	G	S	S	S	S	S	S	S	S
SP033 (145)	V	I	V	V	V	V	V	V	V	V
SP034 (73)	K	N	N	N	N	N	N	N	N	K
SP035 (36)	D	E	E	E	E	E	E	E	E	D
SP036 (308)	M	L	L	L	L	L	L	L	L	M
SP049 (160)	K	R	R	R	R	R	R	R	K	K
SP049 (384)	M	I	I	I	I	I	I	I	I	M
SP049 (596)	D	Y	Y	Y	Y	Y	Y	Y	Y	D
SP054 (405)	F	L	L	L	L	L	L	L	L	F
SP059 (17)	Q	P	P	P	P	P	P	P	P	Q
SP071 (905)	T	I	T	T	T	T	T	T	T	T
SP083 (61)	A	S	S	S	S	S	S	S	A	A
SP097 (348)	S	N	N	N	N	N	N	N	N	S
SP099 (284)	I	N	I	I	I	I	I	I	I	I
SP101 (179)	R	K	K	K	K	K	K	K	K	R
SP115 (116)	D	Y	Y	Y	Y	Y	Y	Y	Y	D
SP115 (340)	K	R	R	R	R	R	R	R	R	K
SP122 (9)	T	N	N	N	N	N	N	N	T	T
SP126 (93)	N	D	D	D	D	D	D	D	D	N
SP126 (135)	S	P	P	P	P	P	P	P	P	S
SP133 (36)	V	I	I	I	I	I	I	I	I	V
SP134 (598)	L	F	F	F	F	F	F	F	L	L
SP134 (1986)	S	R	R	R	R	R	R	R	R	S
SP140 (214)	N	K	N	N	N	N	N	N	N	N
SP143 (16)	T	A	A	A	A	A	A	A	T	T
SP144 (289)	V	L	V	V	V	V	V	V	V	V
SP144 (426)	N	H	H	H	H	H	H	H	H	N
SP149 (85)	L	I	I	I	I	I	I	I	I	L
SP150 (2)	D	N	N	N	N	N	N	N	N	D
SP152 (124)	I	M	I	I	I	I	I	I	I	I

## Data Availability

The reported sequences can be found at GenBank under the accession numbers OQ434235–OQ434239.

## Supplementary Materials

Supplementary Materials Table S1: provides information on the genome nucleotide identity across the compared strains based on a pairwise comparison of SPPV isolates from Genbank and this study.
