# Supplementary material for: Molecular and Epidemiological Analyses of Sheeppox Outbreaks in Russia from 2013 to 2021
Source: Transbound Emerg Dis. 2023 Oct 10;2023:8934280. doi: 10.1155/2023/8934280 (PMC12016853; doi:10.1155/2023/8934280)
Supplement: Supplementary Materials — Table S1: provides information on the genome nucleotide identity across the compared strains based on a pairwise comparison of SPPV isolates from Genbank and this study. [file 8934280.f1.docx]

|  |  | SPPV_Amur_Russia_2018 | KT438551_SPPV_GL_China_2013 | KT438550_SPPV_GH_China_2013 | SPPV_Dagestan_Russia_2022 | SPPV_Pskov_Russia_2019 | SPPV_Twer_Russia_2019 | SPPV_Kostroma_Russia_2020 | ON961657_SPPV_Tula_Russia_2019 | ON961655_SPPV_Moscow_Russia_2018 | ON961656_SPPV_Moscow_Russia_2019 | AY077833_Sheeppox_A_Kazakhstan_2000 | AY077834_SPPV_NISKHI-vaccine | AY077832_Sheeppox_TU-V02127_Turkey_1977 | NC_004002_SPPV_17077-99_Turkey_1970s | MW167071_SPPV_V293_Egypt_2018 | MW167070_SPPV_V123_India_2013 | MW020571_SPPV_V104 | MN072631_SPPV_Turkey-vaccine | MN072630_SPPV_Saudi-Arabia | MT137384_SPPV_Srinagar-P40-vaccine | MN072629_SPV_Pendik | MN072628_SPPV_Nigeria | MN072627_SPPV_Saudi-Arabia-Vaccine | MN072626_SPPV_Abu-Gharib_Iraq | MG000156_SPPV_Jaipur_India_1981 | MG000157_SPPV_Romanian-Fenner_Vaccine |
| --- | --- | --- | --- | --- | --- | --- | --- | --- | --- | --- | --- | --- | --- | --- | --- | --- | --- | --- | --- | --- | --- | --- | --- | --- | --- | --- | --- |
|  |  | 1 | 2 | 3 | 4 | 5 | 6 | 7 | 8 | 9 | 10 | 11 | 12 | 13 | 14 | 15 | 16 | 17 | 18 | 19 | 20 | 21 | 22 | 23 | 24 | 25 | 26 |
| SPPV_Amur_Russia_2018 | 1 |  | 13 | 17 | 219 | 192 | 196 | 195 | 241 | 235 | 233 | 214 | 181 | 405 | 570 | 397 | 325 | 574 | 170 | 188 | 199 | 654 | 355 | 1914 | 1876 | 4912 | 16669 |
| KT438551_SPPV_GL_China_2013 | 2 | 99,99 |  | 6 | 214 | 187 | 191 | 190 | 236 | 230 | 228 | 211 | 178 | 402 | 567 | 394 | 322 | 571 | 167 | 185 | 196 | 651 | 350 | 1909 | 1871 | 4907 | 16663 |
| KT438550_SPPV_GH_China_2013 | 3 | 99,99 | 100 |  | 219 | 193 | 197 | 196 | 242 | 236 | 234 | 215 | 180 | 404 | 569 | 396 | 326 | 573 | 171 | 189 | 200 | 653 | 352 | 1914 | 1876 | 4908 | 16668 |
| SPPV_Dagestan_Russia_2022 | 4 | 99,85 | 99,86 | 99,85 |  | 65 | 67 | 68 | 114 | 106 | 107 | 174 | 154 | 461 | 617 | 449 | 309 | 544 | 148 | 171 | 151 | 706 | 396 | 1975 | 1942 | 4953 | 16716 |
| SPPV_Pskov_Russia_2019 | 5 | 99,87 | 99,88 | 99,87 | 99,96 |  | 4 | 9 | 57 | 51 | 52 | 119 | 104 | 416 | 574 | 402 | 256 | 498 | 99 | 116 | 96 | 659 | 351 | 1918 | 1885 | 4911 | 16678 |
| SPPV_Twer_Russia_2019 | 6 | 99,87 | 99,87 | 99,87 | 99,96 | 100 |  | 9 | 58 | 52 | 53 | 120 | 108 | 419 | 577 | 406 | 260 | 502 | 103 | 120 | 98 | 663 | 349 | 1920 | 1887 | 4912 | 16679 |
| SPPV_Kostroma_Russia_2020 | 7 | 99,87 | 99,87 | 99,87 | 99,95 | 99,99 | 99,99 |  | 61 | 53 | 54 | 123 | 108 | 418 | 578 | 403 | 256 | 501 | 103 | 118 | 101 | 664 | 350 | 1921 | 1887 | 4912 | 16679 |
| ON961657_SPPV_Tula_Russia_2019 | 8 | 99,84 | 99,84 | 99,84 | 99,92 | 99,96 | 99,96 | 99,96 |  | 20 | 19 | 85 | 149 | 443 | 609 | 445 | 299 | 540 | 144 | 161 | 101 | 702 | 391 | 1905 | 1872 | 4876 | 16643 |
| ON961655_SPPV_Moscow_Russia_2018 | 9 | 99,84 | 99,85 | 99,84 | 99,93 | 99,97 | 99,97 | 99,96 | 99,99 |  | 5 | 82 | 143 | 431 | 601 | 433 | 295 | 535 | 138 | 155 | 95 | 692 | 385 | 1901 | 1868 | 4876 | 16645 |
| ON961656_SPPV_Moscow_Russia_2019 | 10 | 99,84 | 99,85 | 99,84 | 99,93 | 99,97 | 99,96 | 99,96 | 99,99 | 100 |  | 81 | 144 | 429 | 599 | 431 | 296 | 534 | 139 | 154 | 94 | 690 | 385 | 1899 | 1866 | 4876 | 16643 |
| AY077833_Sheeppox_A_Kazakhstan_2000 | 11 | 99,86 | 99,86 | 99,86 | 99,88 | 99,92 | 99,92 | 99,92 | 99,94 | 99,95 | 99,95 |  | 121 | 415 | 588 | 418 | 267 | 516 | 107 | 126 | 84 | 675 | 355 | 1880 | 1847 | 4854 | 16621 |
| AY077834_SPPV_NISKHI-vaccine | 12 | 99,88 | 99,88 | 99,88 | 99,9 | 99,93 | 99,93 | 99,93 | 99,9 | 99,9 | 99,9 | 99,92 |  | 394 | 559 | 383 | 190 | 408 | 66 | 84 | 102 | 641 | 324 | 1904 | 1870 | 4884 | 16664 |
| AY077832_Sheeppox_TU-V02127_Turkey_1977 | 13 | 99,73 | 99,73 | 99,73 | 99,69 | 99,72 | 99,72 | 99,72 | 99,7 | 99,71 | 99,71 | 99,72 | 99,74 |  | 213 | 98 | 292 | 525 | 394 | 413 | 414 | 370 | 463 | 2027 | 1993 | 5094 | 16806 |
| NC_004002_SPPV_17077-99_Turkey_1970s | 14 | 99,62 | 99,62 | 99,62 | 99,59 | 99,62 | 99,62 | 99,61 | 99,59 | 99,6 | 99,6 | 99,61 | 99,63 | 99,86 |  | 279 | 448 | 696 | 560 | 580 | 572 | 546 | 630 | 2157 | 2126 | 5035 | 16752 |
| MW167071_SPPV_V293_Egypt_2018 | 15 | 99,74 | 99,74 | 99,74 | 99,7 | 99,73 | 99,73 | 99,73 | 99,7 | 99,71 | 99,71 | 99,72 | 99,74 | 99,93 | 99,81 |  | 277 | 518 | 381 | 400 | 401 | 367 | 458 | 2054 | 2019 | 5098 | 16837 |
| MW167070_SPPV_V123_India_2013 | 16 | 99,78 | 99,79 | 99,78 | 99,79 | 99,83 | 99,83 | 99,83 | 99,8 | 99,8 | 99,8 | 99,82 | 99,87 | 99,81 | 99,7 | 99,82 |  | 338 | 220 | 240 | 255 | 533 | 480 | 2043 | 2008 | 5029 | 16802 |
| MW020571_SPPV_V104 | 17 | 99,62 | 99,62 | 99,62 | 99,64 | 99,67 | 99,66 | 99,67 | 99,64 | 99,64 | 99,64 | 99,66 | 99,73 | 99,65 | 99,54 | 99,65 | 99,77 |  | 470 | 487 | 499 | 252 | 725 | 2299 | 2265 | 5278 | 17056 |
| MN072631_SPPV_Turkey-vaccine | 18 | 99,89 | 99,89 | 99,89 | 99,9 | 99,93 | 99,93 | 99,93 | 99,9 | 99,91 | 99,91 | 99,93 | 99,96 | 99,74 | 99,63 | 99,75 | 99,85 | 99,69 |  | 44 | 101 | 639 | 313 | 1898 | 1861 | 4889 | 16655 |
| MN072630_SPPV_Saudi-Arabia | 19 | 99,87 | 99,88 | 99,87 | 99,89 | 99,92 | 99,92 | 99,92 | 99,89 | 99,9 | 99,9 | 99,92 | 99,94 | 99,72 | 99,61 | 99,73 | 99,84 | 99,67 | 99,97 |  | 116 | 660 | 339 | 1912 | 1875 | 4903 | 16670 |
| MT137384_SPPV_Srinagar-P40-vaccine | 20 | 99,87 | 99,87 | 99,87 | 99,9 | 99,94 | 99,93 | 99,93 | 99,93 | 99,94 | 99,94 | 99,94 | 99,93 | 99,72 | 99,62 | 99,73 | 99,83 | 99,67 | 99,93 | 99,92 |  | 664 | 354 | 1880 | 1851 | 4875 | 16653 |
| MN072629_SPV_Pendik | 21 | 99,56 | 99,57 | 99,56 | 99,53 | 99,56 | 99,56 | 99,56 | 99,53 | 99,54 | 99,54 | 99,55 | 99,57 | 99,75 | 99,64 | 99,76 | 99,64 | 99,83 | 99,57 | 99,56 | 99,56 |  | 714 | 2312 | 2279 | 5360 | 17093 |
| MN072628_SPPV_Nigeria | 22 | 99,76 | 99,77 | 99,76 | 99,74 | 99,77 | 99,77 | 99,77 | 99,74 | 99,74 | 99,74 | 99,76 | 99,78 | 99,69 | 99,58 | 99,69 | 99,68 | 99,52 | 99,79 | 99,77 | 99,76 | 99,52 |  | 1965 | 1929 | 5041 | 16706 |
| MN072627_SPPV_Saudi-Arabia-Vaccine | 23 | 98,72 | 98,73 | 98,72 | 98,68 | 98,72 | 98,72 | 98,72 | 98,73 | 98,73 | 98,73 | 98,74 | 98,73 | 98,65 | 98,56 | 98,63 | 98,64 | 98,47 | 98,73 | 98,72 | 98,74 | 98,46 | 98,69 |  | 381 | 4833 | 15310 |
| MN072626_SPPV_Abu-Gharib_Iraq | 24 | 98,75 | 98,75 | 98,75 | 98,7 | 98,74 | 98,74 | 98,74 | 98,75 | 98,75 | 98,75 | 98,77 | 98,75 | 98,67 | 98,58 | 98,65 | 98,66 | 98,49 | 98,76 | 98,75 | 98,76 | 98,48 | 98,71 | 99,74 |  | 5041 | 15413 |
| MG000156_SPPV_Jaipur_India_1981 | 25 | 96,72 | 96,72 | 96,72 | 96,69 | 96,72 | 96,72 | 96,72 | 96,74 | 96,74 | 96,74 | 96,76 | 96,74 | 96,6 | 96,64 | 96,6 | 96,64 | 96,48 | 96,73 | 96,73 | 96,74 | 96,42 | 96,63 | 96,75 | 96,62 |  | 12206 |
| MG000157_SPPV_Romanian-Fenner_Vaccine | 26 | 88,87 | 88,87 | 88,87 | 88,84 | 88,86 | 88,86 | 88,86 | 88,88 | 88,88 | 88,88 | 88,9 | 88,87 | 88,79 | 88,82 | 88,77 | 88,79 | 88,62 | 88,88 | 88,87 | 88,88 | 88,6 | 88,84 | 89,68 | 89,61 | 91,59 |  |
